# *ZNF76* predicts prognosis and response to platinum chemotherapy in human ovarian cancer

**DOI:** 10.1042/BSR20212026

**Published:** 2021-12-08

**Authors:** Tian Hua, Rui-min Wang, Xiao-chong Zhang, Bei-bei Zhao, Shao-bei Fan, Deng-xiang Liu, Wei Wang

**Affiliations:** 1 Department of Gynaecology, Affiliated Xing Tai People Hospital of Hebei Medial University 399 Shunde Road, Xingtai 054001, China; 2 Department of Clinical laboratory, Affiliated Xingtai People Hospital of Hebei Medial University, 399 Shunde Road, Xingtai 054001, China; 3 Department of oncology, Affiliated Xingtai People Hospital of Hebei Medial University 399 Shunde Road, Xingtai 054001, China; 4 Department of Obstetrics and Gynaecology, Hebei Medical University, Second Hospital, Shijiazhuang 050001, China

**Keywords:** Bioinformatics, OV, Platinum resistance, Prognosis, ZNF76

## Abstract

Ovarian cancer (OV) is the most lethal gynecologic malignancy. One major reason of the high mortality of the disease is due to platinum-based chemotherapy resistance. Increasing evidence reveal the important biological functions and clinical significance of zinc finger proteins (ZNFs) in OV. In the present study, the relationship between the zinc finger protein 76 (*ZNF76*) and clinical outcome and platinum resistance in patients with OV was explored. We further analyzed *ZNF76* expression via multiple gene expression databases and identified its functional networks using cBioPortal. RT-qPCR and IHC assay shown that the ZNF76 mRNA and protein expression were significantly lower in OV tumor than that in normal ovary tissues. A strong relationship between *ZNF76* expression and platinum resistance was determined in patients with OV. The low expression of *ZNF76* was associated with worse survival in OV. Multivariable analysis showed that the low expression of *ZNF76* was an independent factor predicting poor outcome in OV. The prognosis value of *ZNF76* in pan-cancer was validated from multiple cohorts using the PrognoScan database and GEPIA 2. A gene-clinical nomogram was constructed by multivariate cox regression analysis, combined with clinical characterization and *ZNF76* expression in TCGA. Functional network analysis suggested that *ZNF76* was involved in several biology progressions which associated with OV. Ten hub genes (*CDC5L*, *DHX16*, *SNRPC*, *LSM2*, *CUL7*, *PFDN6*, *VARS*, *HSD17B8*, *PPIL1*, and *RGL2*) were identified as positively associated with the expression of *ZNF76* in OV. In conclusion, *ZNF76* may serve as a promising prognostic-related biomarker and predict the response to platinum in OV patients.

## Introduction

Ovarian cancer (OV) is the 7th most common cancer in women, with an estimated 239 000 new cases and 152 000 deaths worldwide annually [1]. The majority of patients are initially diagnosed with advanced-stage disease due to the lack of early specific symptoms. Platinum-based chemotherapy is the first-line treatment following primary cytoreductive surgery for advanced-stage OV patients. Despite showing an optimal response to this initial regimen, most patients will suffer from relapse with platinum-resistant tumors within 24 months. The 5-year survival rate for OV patients with the advanced-stage disease is still only approximately 30% [2]. Platinum resistance, either primary or acquired, is the main obstacle to successful treatment and remains a major problem in the management of patients with OV. Multiple studies have shown that transcriptional variations can play an important role in regulating the platinum resistance [3–5].

Transcription factors (TFs) recognized as the key regulators of gene expression are involved in a large number of cancers for which they account for approximately 20% of all oncogenes identified so far. TFs are considered to be crucial for all development processes in tumorigenesis, metastasis, and therapy [6]. It is necessary to deeply understand these transcription factors and their target genes, which may provide the possibility of the new therapies. The zinc fingers proteins (ZNFs) are the largest family of DNA-binding proteins and can act as TFs in eukaryotes, which conduct various molecular functions, including development, differentiation, metabolism, and autophagy [7]. Over the last few decades, increasing evidence reveal the important biological functions and clinical significance of ZNFs in OV [8]. Some ZNFs act as tumor suppressors, while others act as tumor oncogenes. Various studies have shown that ZNFs were associated with ovarian cancer cell growth and invasive ability, cell proliferation and metastasis, survival time in OV patients, clinical stage, and poor prognosis [9–12].

Zinc finger protein 76 (*ZNF76*), first identified in 1991, locates on chromosome 6p21 and belongs to a member of the GLI-Kriippel family of DNA-binding proteins [13]. ZNF76 is a human homolog of Staf (selenocysteine tRNA gene transcription-activating factor) [14], which is a Xenopus zinc-finger transcription factor known to regulate genes encoding selenocysteine tRNA (tRNAsec) and small nuclear RNA. ZNF76 was also shown to activate transcription of a molecular chaperonin subunit Ccta gene [15], suggesting multiple roles of ZNF76 for transcriptional regulation. Early studies implicated that ZNF76 could target TATA-binding protein and repress the transcription [14]. Studies have shown *ZNF76* is associated with a range of phenotypic abnormalities that affect embryonic development, male fertility, and neoplasia [16,17]. However, few pertinent studies associated with *ZNF76* in cancers have been reported.

The present study is to investigate the relationship between the zinc finger protein 76 (ZNF76) and clinical outcome and platinum chemotherapy resistance in patients with OV. Furthermore, we visualized the prognostic landscape of *ZNF76* in pan-cancer using databases, including PrognoScan, GEPIA 2, and Kaplan–Meier Plotter. The functional networks related to *ZNF76* in OV were also evaluated.

## Materials and methods

### Tissue samples

Tissue samples of OV patients were collected from 85 patients at the time of the first surgery between May 2016 and May 2018. Inclusion criterion involved the histopathological diagnosis of OV and received cytoreductive debulking surgery followed platinum-based chemotherapy as well as no treatment prior to surgery. Patients who do not meet the above criterion were excluded. Patient characteristics are listed in Table 1. Based on the platinum-free interval (PFI), the 85 OV patients were divided into a platinum-resistant group (*n*=35) and a platinum-sensitive group (*n*=50). A PFI of <6 months was considered to platinum resistance, whereas a PFI of >6 months was considered to platinum sensitivity [18]. The participants were followed up regularly for more than 3 years. Progression-free survival (PFS) and overall survival (OS) were used to analyze the survival status of OV patients.

**Table 1:** Clinical characteristics of patients with ovarian cancer

Characteristic	Platinum-based chemotherapy		*P*-value
	Sensitive group (*n*=50)	Resistant group (*n*=35)	
**Age (year)**			
≤50	20	11	0.419
>50	30	24	
**Stage**			
I-II	12	6	0.446
III-IV	38	29	
**Grade**			
G1	16	5	0.152
G2	17	17	
G3	17	13	
**Pathology**			
Serous	38	25	0.638
Endometrioid	12	10	
**Tumor size**			
<10 cm	36	28	0.400
≥10 cm	14	7	

Abbreviation: FIGO, International Federation of Gynecology and Obstetrics.

Thirty normal ovarian epithelial tissues were collected from the patients who underwent the adnexectomy for gynecological benign diseases at the same time. The study was approved by the Ethics Committee of Affiliated Xingtai People Hospital of Hebei Medial University (2021 [011]). All patients provided written informed consent.

**Figure 1:**
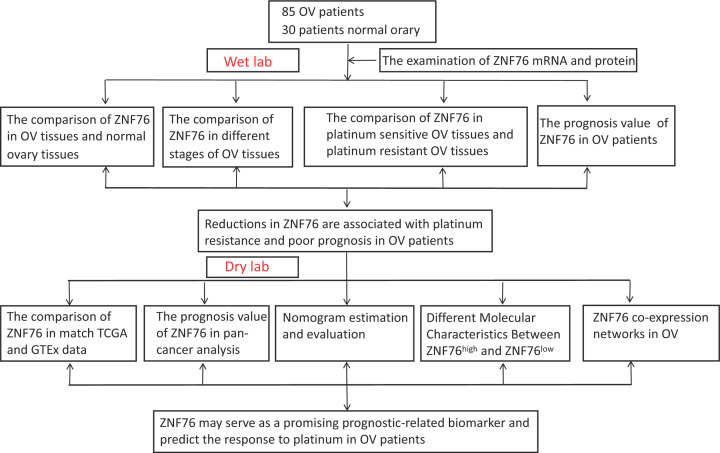
Schematic diagram representation of the study design

A two stage (wet lab and dry lab) schematic diagram of the study design was shown in Figure 1.

### Reverse transcription and real-time quantitative PCR (RT-qPCR)

Total RNA was extracted using TRIzol reagent (Generay Biotech, Shanghai, Co., Ltd., China), according to manufacturer’s protocol. Revert-Aid First Strand cDNA Synthesis Kit (Thermo Scientific, U.S.A.) was used to synthesize cDNA from 500 ng total RNA. Reverse transcription quantitative PCR was done using QuantiNova TMSYBR® Green PCR Kit (Qiagen, Hilden, Germany). The primer sequences were obtained from Sangon Biotech Co. Ltd (Table 2) (Shanghai, China). GAPDH was used as housekeeping gene. Each sample was measured in triplicate.

**Table 2:** The primer sequences of genes

Gene	The primer sequences
ZNF76-F	GTGATGGGACAACAGCCTACG
ZNF76-R	GCTTCTTTCTGTACCGTCACC
CDC5L-F	GAAGGTCGCGCTTGGAGGAA
CDC5L-R	CAGGGTGTCTCGGCAAGCAG
DHX16-F	TGTGCGGGAACAGCTGGAAG
DHX16-R	GTCAACCGTGCCGTGTGGTA
SNRPC-F	ATGATGGGCCCTCCTCCTCC
SNRPC-R	CTGGTCGAGTCATTCCGGGC
LSM2-F	TGCTACAGGATGCGGCAAGG
LSM2-R	TGGGGGTTAGGGGTTCTGGG
CUL7-F	TGGGCCCCTCACCTCTTCAA
CUL7-R	TCTGGCCGTCTTCACCCTCA
PFDN6-F	TGTCTCGCACCCAGTAGGCT
PFDN6-R	TCTGCCTCCCCGACATGGAT
VARS-F	CGCTCCCTGTCACAAAGGGG
VARS-R	CGGGAATCCACCTCACAGCC
HSD17B8-F	GCCCTGGTGTCCAATGGTTGT
HSD17B8-R	AGCGGATCCCATGTCGTCCA
RGL2-F	CATCCGCAATCTCCGGTCCC
RGL2-R	GGTCAGCTGTTCGGCCAAGT
PPIL1-F	TGACCCAACAGGGACAGGTC
PPIL1-R	GTTCATCTTCAAACTGTTTGCCAT
GAPDH-F	GACCTGACCTGCCGTCTA
GAPDH-R	AGGAGTGGGTGTCGCTGT

### ZNF76 immunohistochemistry study of clinical samples

Of the 85 OV and 30 normal ovary tissue samples, 28 OV and 21 normal ovary tissue samples were obtained in the pathology department of the Affiliated Xingtai People Hospital of Hebei Medial University. The expression of ZNF76 was evaluated with immunohistochemical (IHC) staining. Rabbit antihuman ZNF76 (YT4982, 1:300 dilution; ImmunoWay Biotechnology Co., Ltd., U.S.A.) was applied to detect ZNF76. Immunoreactivity for ZNF76 was considered positive in cells showing nuclear staining without cytoplasmic staining. The expression of the ZNF76 protein was evaluated based on https://www.proteinatlas.org [19]. Specifically, negative staining (no nuclear staining of any cells), weak expression (nuclear staining of <25% of cells), moderate expression (nuclear staining of 25–75% of cells), and strong expression (nuclear staining of >75% of cells).

### Data retrieving and pre-processing

The mRNA expression profiles and clinical features was obtained from the TCGA database and GTEX database, which was downloaded through the University of California Santa Cruz Xena (UCSC Xena; https://xena.ucsc.edu/) [20] platform. The TCGA data included 60484 genes, and GTEX data included 60499 genes annotated by GENCODE version 23. A custom Perl script was used to extract the data from Ovary cancer from GTEX data for subsequent analysis; GTEX data was re-calculated from log2 (*x*+0.001) transformed to log2 (*x*+1) transformed, and then the statistical Wilcoxon rank-sum test was performed to compare the differential expression of *ZNF76*.

### GEPIA2 analysis

Gene Expression Profiling Interactive Analysis 2 (GEPIA2) [21] is a web server for analyzing the RNA sequencing expression data of 9736 tumors and 8587 normal samples from the TCGA and the GTEx projects (http://gepia2.cancer-pku.cn). The differential expression of *ZNF76* in cancer and normal tissues for 33 cancer types was analyzed using the ‘Dot plot’ function of GEPIA2. The expression of a gene is plotted in log2 (TPM + 1) scale, in which TPM is the Transcript Per Million value. Analysis of variance (ANOVA) was used to test the statistical significance of the difference of expression in tumor tissues versus paired normal tissues.

### Heatmaps, clustering analysis, and differentially expressed genes

We divided the pre-processing expression of *ZNF76* downloaded from of UCSC Xena data into high and low groups by median. Differentially expressed genes between the two groups were analyzed using the Wilcoxon rank-sum test. To express the results of differentially expressed gene (DEG) screening and cluster analysis, |log (FC)| > log1.5 and FDR <0.05 were set in performing heatmaps based on a heatmap R package and in performing volcano plots based on a ggplot2 R package.

### PrognoScan database analysis

The correlation between *ZNF76* expression and survival in various types of cancers was analyzed by the PrognoScan database (http://www.abren.net/PrognoScan/) [22]. The threshold was adjusted to a Cox *P*-value < 0.05.

### Construction and assessment of the nomogram

Univariate and multivariate cox regression analysis was done to identify the proper terms to build the nomogram based on pre-processing expression of ZNF76 downloaded from of UCSC Xena data. The forest plot was used to show the *P*-value, HR, and 95% CI of each variable through the ‘forestplot’ R package. We use the ‘rms’ package from R to perform the nomogram, calibration plots. Nomogram was used as a prediction to evaluate the prognosis of patients, which can generate an individual probability of a clinical event by integrating various prognostic factors. Calibration was performed to evaluate the performance of the 3- and 5-year OS nomogram. The *x*-axis indicated nomogram-predicted survival, and the *y*-axis represented observed outcome, and the 45° line represented the best prediction.

### Gene set enrichment analysis (GSEA)

According to the median value of pre-processing expression of ZNF76 downloaded from of UCSC Xena data, we divided the samples into high- and low-*ZNF76* groups. GSEA was used to explore the biological function of prognostic signature for two groups. Annotated gene sets of c5.go.v7.2.symbols.gmt in the Molecular Signatures Database (MSigDB) was were chosen as the reference gene sets by GSEA version 4.0.3 [23]. We performed 1000 times of permutations. The collapsed dataset to gene symbols was ‘False’. The permutation type was ‘phenotype’. GSEA was performed, and the cut-off criteria were as follows: false discovery rate (FDR) *q* < 0.25 and nominal *P*<0.05.

### Protein interaction network (PPI) and pathways interaction analysis building

We identified genes co-expressed with *ZNF76* using the cBioPortal database (http://www.cbioportal.org) [24]. A Spearman's correlation coefficient exceeding 0.30 indicated a good correlation between *ZNF76* and a co-expressed gene. SRTING version 11.0 [25] was used to evaluate the PPI information of the co-expression genes of *ZNF76*, and their biological functions were also obtained with a minimum interaction score cutoff of 0.4 and hide disconnected nodes in the network. Then, the interaction network of these proteins was visualized by Cytoscape3.7.2 [26], and the CytoHubba plug-in was used to identify hub genes with the criteria of filtering degree ≥10.

### Statistical analyses

R software v3.6.3 (R Foundation for Statistical Computing, Vienna, Austria) was used in statistical analyses. Fisher’s exact test or Pearson’s chi-square test was used for analyze qualitative variables, and Wilcoxon rank-sum test (for unpaired samples) was used for quantitative variables. Survival analysis was done by Kaplan–Meier analysis. The Kruskal–Wallis test was applied for normalizing multiple groups. If not specified above, *P*<0.05 was considered with statistical significance.

## Results

### Expression of *ZNF76* is down-regulated in OV tissues

**Figure 2:**
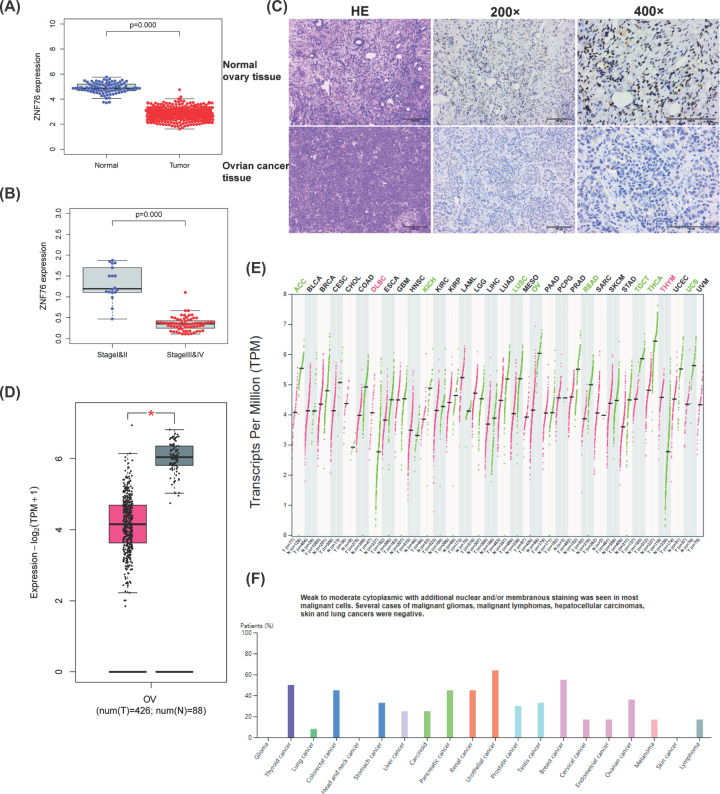
The expression *ZNF76* in ovarian cancer (OV) (**A**) The comparison of *ZNF76* expression between OV tumor tissue and normal ovary tissue. (**B**) The comparison of *ZNF76* expression between early stage patients and advanced stage patients. (**C**) The comparison of ZNF76 protein expression between OV tumor tissue and normal ovary tissue. (**D**) Transcriptional level of *ZNF76* expression was found lowly expressed in 426 ovarian cancer (OV) tissues compared with 88 normal tissues in TCGA cohort (*P<0.01). (**E**) Differential *ZNF76* expression in cancer tumor tissues and normal tissues based on TCGA and GTEX database. (**F**) The ZNF76 protein expression in multiple cancer types based on data from the human protein atlas.

Archived information of 85 OV patients was obtained from the Affiliated Xingtai People Hospital of Hebei Medial University. The 30 normal ovarian epithelial tissues were collected from the patients who underwent the adnexectomy for gynecological benign diseases. The median age was 61 years old (age ranges from 46 to 79). They had no previous diagnosis of cancer. RT-qPCR was performed to investigate the mRNA expression of *ZNF76* in OV tissues and normal ovary tissues. The expression levels of *ZNF76* mRNA in OV tissues were significantly lower than those from normal ovary tissues (Figure 2A, *P*<0.001). The expressions of *ZNF76* in stage III–IV were lower than those in stages I–II OV (Figure 2B, *P*<0.001). Compared with the normal ovary tissues, the expression of the ZNF76 protein was significantly decreased in the OV tissues (Figure 2C and Table 3, *P*=0.013). Further, the *ZNF76* mRNA expressions from TCGA samples and the GTEx projects were compared using the GEPIA 2. The *ZNF76* expression in 426 OV tumors compared with 88 normal tissues is shown in Figure 2D, and statistically significant differences were observed (*P*<0.001). Therefore, ZNF76 may serve as a tumor suppressor in OV. However, the role of ZNF76 in pan-cancer may be different. The differential expression between the tumor and normal tissues for ZNF76 across the different tumors is shown in Figure 2E. The expression of ZNF76 protein in OV samples was found to be higher than the median in pan-cancer analysis based on the data from the human protein atlas (Figure 2F).

**Table 3:** ZNF76 protein expression differences between the ovarian cancer tissues and the normal ovary tissues

ZNF76 expression	Ovarian cancer tissues, *n* (%)	Normal ovary tissues, *n* (%)	*P-*value
Negative	7 (25.00)	0 (0.00)	0.013
Weak	17 (60.71)	11 (52.38)	
Moderate	4 (14.28)	8 (38.09)	
Strong	0 (0.00)	2 (9.52)	

### Reductions in *ZNF76* are associated with platinum resistance and poor prognosis in OV patients

**Figure 3:**
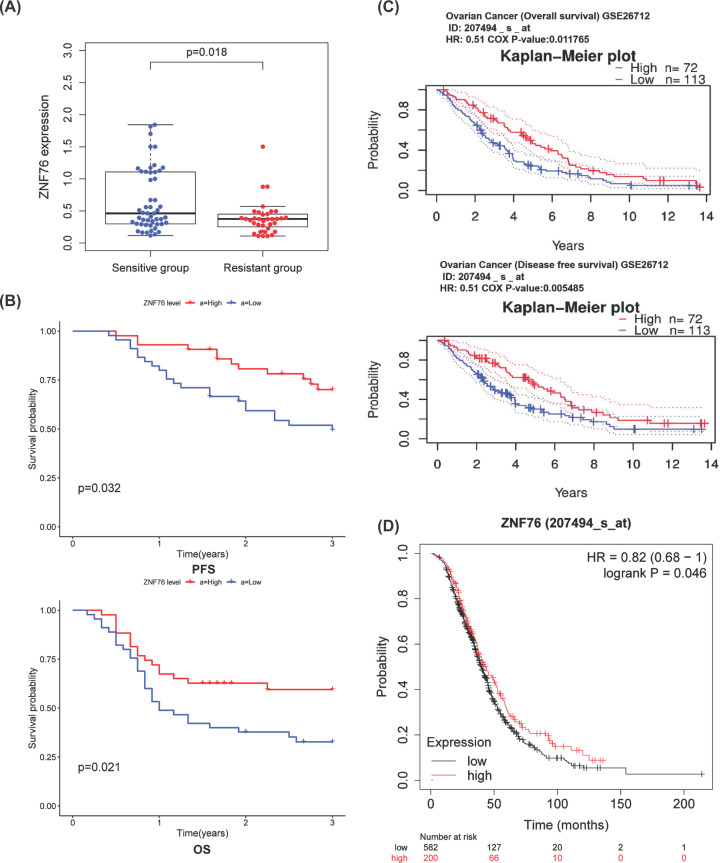
The association between *ZNF76* expression and platinum resistance and ovarian cancer (OV) patients’ prognosis (**A**) *ZNF76* mRNA expression was lower in platinum-resistant patients compared with platinum-sensitive patients (*P*<0.05). (**B**) Kaplan–Meier survival analysis of OV patients with respect to the *ZNF76* expression. (**C**) Kaplan–Meier survival curves comparing the high and low expression of *ZNF76* in OV patients using Kaplan–Meier plotter data. (**D**) Kaplan–Meier survival curves comparing the high and low expression of ZNF76 by PrognoScan Database Analysis.

**Figure 4:**
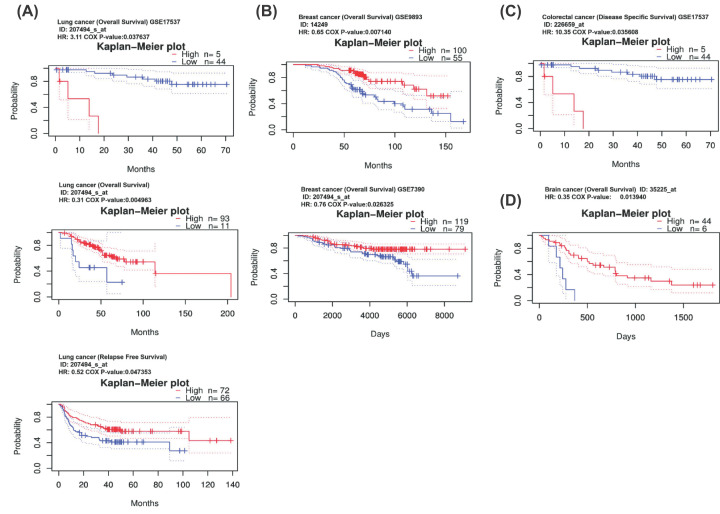
Kaplan–Meier survival curves comparing the high and low expression of ZNF76 in different types of cancer (**A**) Lung cancer, (**B**) breast cancer, (**C**) colorectal cancer, and (**D**) brain cancer.

We have listed the clinicopathological features of the platinum sensitive group and platinum resistant group in Table 1, and there was no difference in the characteristics of the two groups statistically. We investigated the expression levels of *ZNF76* in platinum-resistant and platinum-sensitive OV tissues by RT-qPCR. The levels of ZNF76 were significantly lower in platinum-resistant patients than in platinum-sensitive patients (*P*=0.018, Figure 3A). The 85 patients were divided into low and high groups based on the median value of *ZNF76* expression. A significant relationship was observed between the mRNA expression of *ZNF76* and clinical prognosis in OV patients using Kaplan–Meier analysis. Compared with those patients with *ZNF76*^high^, *ZNF76*^low^ OV patients had shorter PFS (Figure 3B, *P*=0.032) and OS (*P*=0.021). After adjusting for other prognostic factors (age, stage, grade, and tumor residual size), low *ZNF76* expression was also significantly associated with shorter PFS and OS (*P*=0.027, *P*=0.038; Table 4), demonstrating that *ZNF76* expression is an independent predictor of poorer clinical outcome in patients with OV. The results suggested *ZNF76* may act as tumor suppressor in OV. Further, the cohort GSE26712 included 185 OV patients at different stages and showed that low *ZNF76* expression was significantly associated with shorter disease-free survival (DFS) and OS (DFS: Cox *P* = 0.005, HR = 0.51; OS: Cox *P* = 0.011, HR = 0.51, Figure 3C). A significant relationship was also observed between the mRNA expression of *ZNF76* and clinical prognosis in OV patients using Kaplan–Meier plotter data (Figure 3D). The impact of *ZNF76* expression on survival rates of pan-cancer was evaluated using the PrognoScan. Notably, the low expression of *ZNF76* was also found strongly correlated to poor survival in colorectal cancer, brain cancer, breast cancer, and lung cancer (Figure 4A–D). We further added a Supplementary Table S1 to present the pan-cancer survival analysis of ZNF76.

**Table 4:** Prognostic factors in patients with ovarian cancer using the Cox proportional hazards model

	Recurrence			Survival		
	HR	95%CI	*P*-value	HR	95%CI	*P*-value
**Age**						
≤50 vs. >50	1.150	0.72–1.82	0.551	0.988	0.59–1.64	0.963
**FIGO stage**						
I–II vs. III–IV	1.066	0.31–3.66	0.919	1.056	0.13–2.74	0.518
**Grade**						
G3 vs. G1-2	0.798	0.49–1.28	0.349	0.680	0.60–1.84	0.849
**Tumor residual size**						
0 cm vs. ≤1 cm vs. >1 cm	4.365	2.78–6.83	0.000	5.185	2.94–9.13	0.000
**Tumor size**						
≤10 cm vs. >10 cm	1.312	0.83–2.06	0.241	1.178	0.70–1.95	0.527
***ZNF76* expression**						
Low vs. high	1.689	1.06–2.69	**0.027**	1.731	1.03–2.90	**0.038**

### Nomogram estimation and evaluation

**Figure 5:**
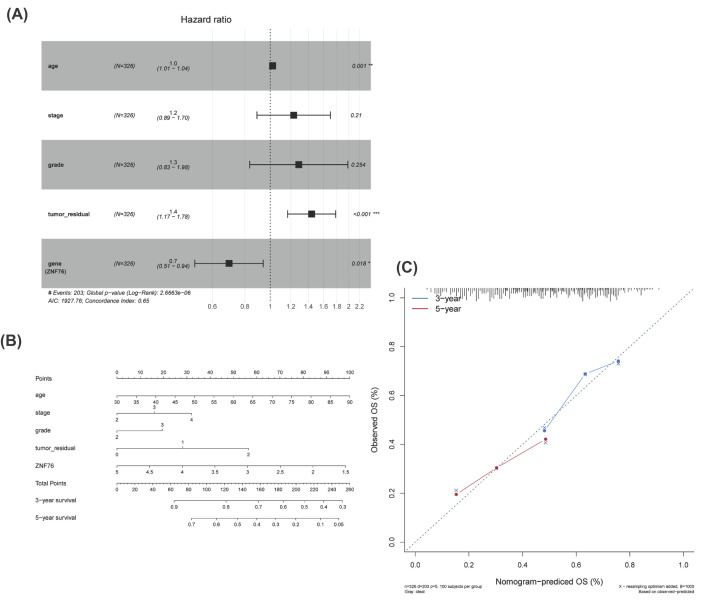
The association between ZNF76 expression and ovarian cancer (OV) patients’ prognosis by bio-informatics analysis (**A**) Forest plots for multivariate Cox analysis. (**B**) Nomogram predicting 3- and 5-year OV survival. (**C**) Calibration curves. Calibration curves for 3- and 5-year cancer specific survival probability depict the calibration of each model. Dashed line represents the ideal nomogram; solid line represents the performance of current nomogram.

To further examine the prognostic-associated role of *ZNF76* in OV patients, the *ZNF76* expression index was included in the multivariable analysis for survival in TCGA. The results showed that *ZNF76* mRNA levels were significantly associated with OS when age, stage, grade, and tumor residual size were included in the model (*P*=0.018, HR = 0.7, 95%CI = 0.51–0.94, Figure 5A). These analyses indicated that *ZNF76* mRNA expression might serve as an independent prognostic biomarker for OV patients. Subsequently, we constructed a gene-clinical nomogram by multivariate cox regression analysis, combined with clinical characterization and *ZNF76* expression (Figure 5B). Nomograms can visually predict the prognosis of patients according to their genes and clinical information, and accurately predict the survival of patients at 3 and 5 years. The predicted values by the nomogram for 3- or 5-year survival probabilities were very close to the observed rate suggesting a strong power of this model (Figure 5C).

### Different molecular characteristics between *ZNF76*^high^ and *ZNF76*^low^

**Figure 6:**
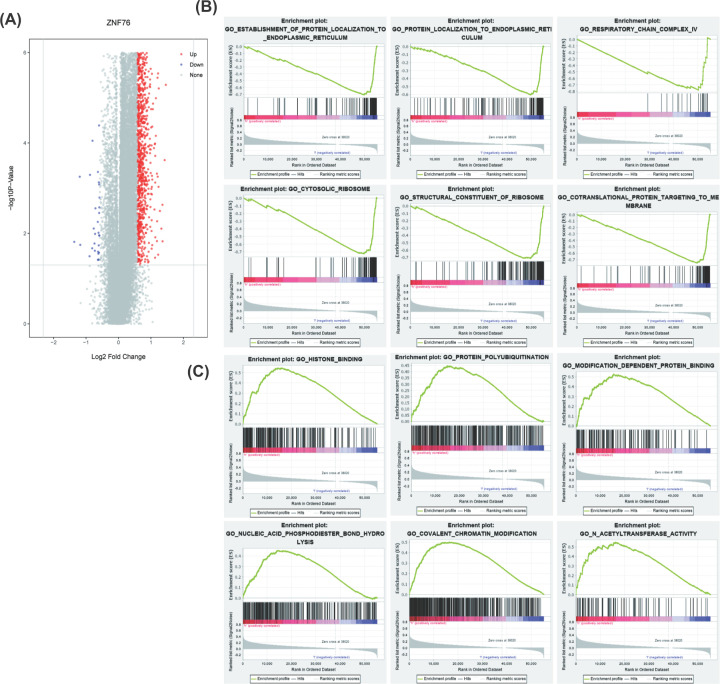
Genome-wide genes and cell signaling pathways associated with *ZNF76* expression (**A**) Volcano plot of differential gene profiles between *ZNF76*^high^ and *ZNF76*^low^. (**B** and **C**) The significantly enriched GO annotations between *ZNF76*^high^ and *ZNF76*^low^ expression in OV were analyzed using GSEA.

To further investigate the biological role of *ZNF76* in OV, the gene expression profiles associated with *ZNF76* were derived based on RNA-seq data. According to the median expression of *ZNF76*, we divided the samples into high- and low- *ZNF76* groups. The results of differential gene profiles were presented as volcano plots between *ZNF76***^high^** and *ZNF76***^low^** (Figure 6A). Further, we identify functions and biological pathways between *ZNF76*^high^ and *ZNF76*^low^ expression on TCGA expression data sets by GSEA (false discovery rate (FDR) *q*<0.25 and nominal *P*<0.05). This analysis showed that the *ZNF76*^low^ phenotype was enriched of many essential biological progressions, such as protein localizing to the endoplasmic reticulum, protein targeting to the plasma membrane, co-translational protein targeting to membrane, and cytosolic ribosome. The histone binding, acetyltransferase activity, and microtubule-based transport were enriched in the *ZNF76***^high^** phenotype (Figure 6B,C). These enhancement or attenuation of these biology progressions might explain the contribution of *ZNF76* on the OV patients’ prognosis.

### *ZNF76* co-expression networks in OV

**Figure 7:**
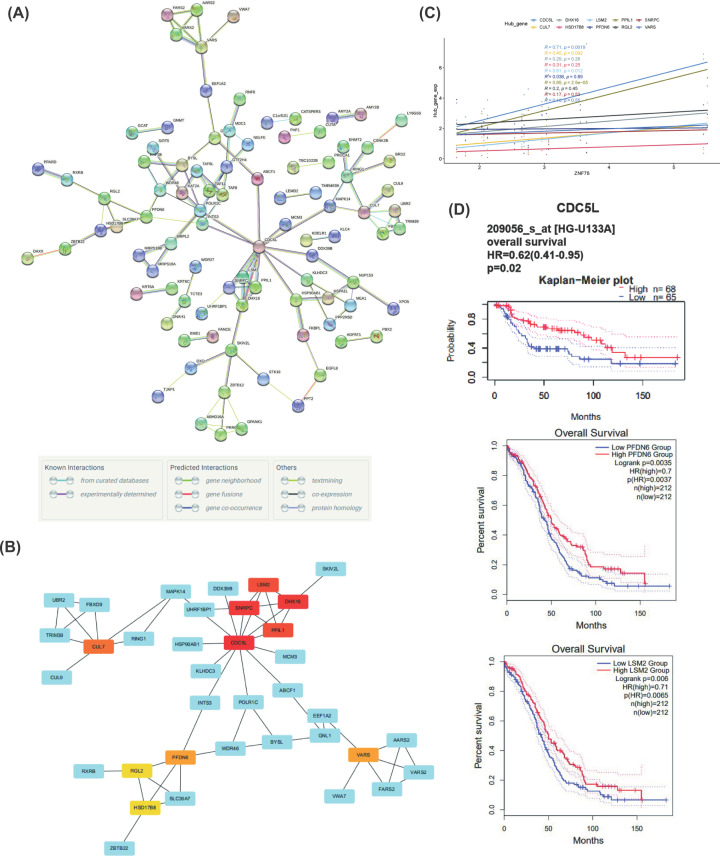
The PPI network of *ZNF76* (**A**) A network of *ZNF76* and its co-expression genes was set up visually using STRING in OV. (**B**) The hub genes (*CDC5L, DHX16, SNRPC, LSM2, CUL7, PFDN6, VARS, HSD17B8, PPIL1*, and *RGL2*) associated with *ZNF76* were identified in OV. (**C**) The correlation analysis between the expressions of the hub genes and *ZNF76* in OV tissues. (**D**) The prognostic value of hub genes (*CDC5L, LSM2*, and *PFDN6*) in OV patients (PrognoScan database).

To gain a deep understanding of *ZNF76* biological functions in OV, the function module of cBioPortal to examine *ZNF76* co-expression mode in the OV cohort. A network of *ZNF76* and its co-expression genes using the STRING online database is shown in Figure. 7A. In total, 257 essential genes that correlate to *ZNF76* expression were identified (Supplementary Table S2). Ten hub genes (*CDC5L, DHX16, SNRPC, LSM2, CUL7, PFDN6, VARS, HSD17B8, PPIL1*, and *RGL2*, Figure 7B) were identified according to the method MCC in CytoHubba. We get these predicted hub genes by 12 methods in cytohubba, due to the limited sets of 5 in Venn diagram, so we selected 5 methods to show the overlapped results in the Venn diagram, seven of ten genes overlapped (Supplementary Figure S1). Venn diagram analysis was performed using the OmicStudio tools at https://www.omicstudio.cn/tool. We further validated the correlations between the expressions of these hub genes with ZNF76 in OV samples. As shown in Figure 7C, the expression of the *CDC5L, PPIL1*, and *LSM2* were positive correlated with the *ZNF76* expression in OV tissues, respectively. However, another hub genes were not present the obvious associations with *ZNF76*, maybe due to the limited samples.

Further survival analysis was performed on these hub genes to evaluate their effects on OV patients’ survival by the GEPIA 2 and PrognoScan. The expressions of *CDC5L*, *LSM2*, and *PFDN6* were clearly related to OV patient prognosis (Figure 7D). The lower expressions of *CDC5L*, *LSM2*, and *PFDN6* indicated a shorter OS compared with the higher group. In addition, we have performed the survival analysis of ten hub genes on pan-cancers based on the PrognoScan. The effect of these hub genes on clinical outcome were inconsistent in the pan-cancer analysis (Supplementary Table S3), which still need to be explored deeply.

## Discussion

The classical ZnF proteins form the largest family of sequence-specific DNA-binding proteins are encoded by 2% of human genes [27,28]. Although some members of the ZnF proteins were demonstrated to be carcinogenic in many neoplasms, the gene expression profile and prognostic value of *ZNF76* in OV were still not elucidated. In the present study, our results confirmed that the expression of ZNF76 was significantly lower in OV tumor compared to that in normal ovary tissues. The expression of *ZNF76* is significantly lower in platinum-resistant tissue compared with platinum-sensitive tissue in OV patients. The lower expression of *ZNF76* was associated with the poorer clinical outcome of patients with OV. The nomogram risk score based on ZNF76 expression combined with clinical characterization as a method to predict prognosis provides a visual method for predicting OS in OV patients. Moreover, the prognosis value of *ZNF76* was validated in pan-cancers through the bioinformatics analysis from public data.

*ZNF76* is a human homolog of Staf (selenocysteine tRNA gene transcription-activating factor) [29]. Additionally, Zheng [14] pointed out *ZNF76* is a novel transcriptional repressor that targets TATA-binding protein [30], leading to repression of p53-mediated transactivation and down-regulation of endogenous p53 target genes. *ZNF76* was shown the strong inhibitory effect on p53 in various cell lines, containing the HeLa, MCF-7, U2OS, and H1299. In the present study, we found that ZNF76 was decreased expressed in OV tumor tissue compared with normal ovary tissues. Therefore, we speculated that the decreased expression of *ZNF76* promoted the pathogenesis of OV, which might be due to de-regulated p53 expression.

Furthermore, our results show that mRNA levels of *ZNF76* are significantly lower in platinum-resistant tissue than in platinum-sensitive tissue. These results provide strong evidence that low expression of *ZNF76* may have a critical role in the platinum chemotherapy resistance in OV. Furthermore, patients with *ZNF76*^low^ expression had a poorer prognosis than those with *ZNF76*^high^ expression. Thus, the deregulation of *ZNF76* expression may significantly increase patients’ chemotherapy resistance to first-line platinum drugs, resulting in a unfavorable prognosis. We also demonstrated that low expression of *ZNF76* mRNA is associated with a poor survival rate in patients with OV for the first time. These results provide strong evidence that the down-regulation of *ZNF76* may contribute to platinum resistance in OV. In addition, we visualized the prognostic landscape of *ZNF76* in pan-cancer using databases. Compared with a high expression level, a low expression level of *ZNF76* was also correlated with a poorer prognosis in breast cancer and lung cancer.

To further explore the possible mechanism of *ZNF76* in OV pathogenesis and drug resistance, GSEA was used to detect its genetic enrichment. GSEA analysis between *ZNF76*^high^ and *ZNF76*^low^ expression on TCGA expression data indicated that *ZNF76*^low^ phenotype was hugely enriched in many significant biological functions correlated with tumorigeneses, such as co-translational protein targeting to membrane, cytosolic ribosome, and respiratory chain complex, nuclear-transcribed mRNA catabolic process nonsense mediated decay. Moreover, we also examined the *ZNF76* PPI network to discover the potential signaling events that correlates to the differential *ZNF76* expression. Ten hub genes (*CDC5L*, *DHX16*, *SNRPC*, *LSM2*, *CUL7*, *PFDN6*, *VARS*, *HSD17B8, PPIL1*, and *RGL2*) were identified to be positively associated with *ZNF76* in OV. However, the prognosis value of these genes in pan-cancers was inconsistent based on the prognoscan datasets. It was still needed to explore in further experiments. Among them, the low expression of *CDC5L*, *LSM2*, and *PFDN6* were statistically more likely to have shorter OS in patients with OV. *CDC5L* was found to act as a candidate oncogene in osteosarcoma [31], cervical tumors, and bladder cancer [32]. *LSM2* was also found to play a role in pre-mRNA splicing via participating the assembling of spliceosome [33,34]. *PFDN6* might serve as a potential biomarker of prognosis and chemotherapy in childhood ALL [35]. This supportive information enhances the understanding of *ZNF76* act as potential biomarkers for diagnosis as well as prognosis. However, the detailed mechanisms of *ZNF76* associated signaling pathways in OV remain unclear. Therefore, future research is required in further experiments.

In conclusion, the present study is the first to explore the expression and prognostic value of *ZNF76* in OV patients. Our results in clinical samples suggested the low expression of *ZNF76* was associated with platinum resistance and could be an adverse biomarker for OV. Moreover, the prognosis value of *ZNF76* in pan-cancer was validated from multiple cohorts. *ZNF76*^low^ phenotype carried various kinds of molecular events. Taken together, *ZNF76* plays an important role in OV and will be potentially a promising prognostic-related biomarker and predict the response to platinum in OV patients. Therefore, our study highlights the potential function of *ZNF76* in OV.

## Supplementary Material

Supplementary Figure S1

Supplementary Table S1-S3

## Data Availability

All datasets generated for this study are included in the Supplementary material.

## Abbreviations

DFS: disease-free survival
IHC: immunohistochemical
OS: overall survival
PFI: platinum-free interval
PFS: progression-free survival
TF: transcription factor
ZNF: zinc finger protein
