# Supplementary material for: ZNF76 predicts prognosis and response to platinum chemotherapy in human ovarian cancer
Source: Biosci Rep. 2021 Dec 8;41(12):BSR20212026. doi: 10.1042/BSR20212026 (PMC8661506; doi:10.1042/BSR20212026)
Supplement: Supplementary Figure S1 [file BSR-2021-2026_supp.pdf]

overlap between predicted hub genes

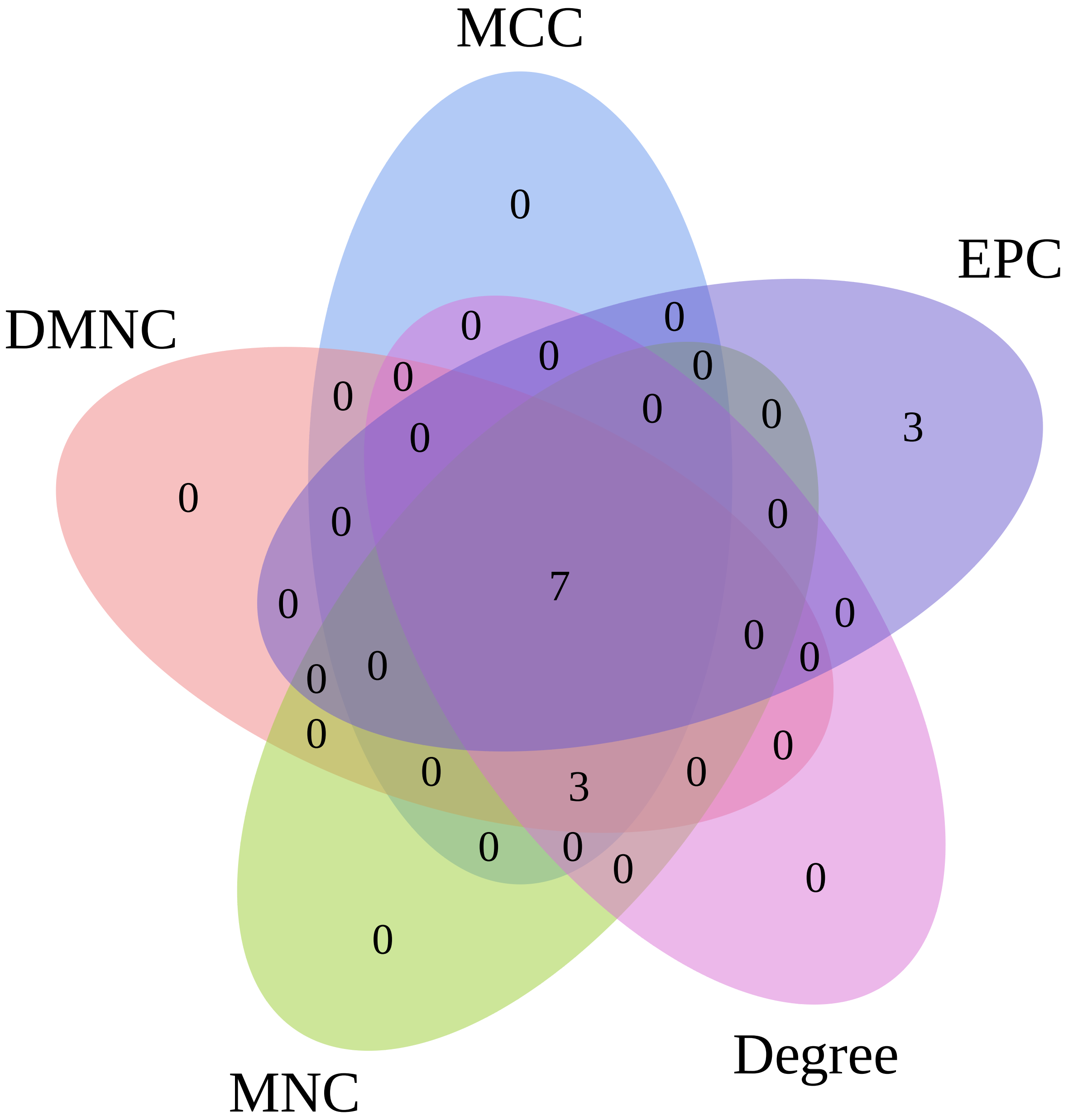

Supplement figure 1 A Venn diagram to show the overlap between predicted hub genes
